# Global trends and academic landscapes of AI applications in basal cell carcinoma research: a bibliometric analysis

**DOI:** 10.3389/fonc.2026.1779358

**Published:** 2026-05-11

**Authors:** Yicheng Li, Yanping Bai, Lina Asihaer

**Affiliations:** 1 Beijing University of Chinese Medicine, School of China-Japan Friendship Hospital Clinical Medicine, Beijing, China; 2 China-Japan Friendship Hospital, Department of Dermatology, Beijing, China

**Keywords:** artificial intelligence, basal cell carcinoma, bibliometrics, deep learing, dermoscopy, machine learning

## Abstract

**Background:**

Basal cell carcinoma (BCC), one of the most prevalent skin cancers, still faces substantial challenges in timely diagnosis and optimal management. Artificial intelligence (AI) holds promise for improving early detection, risk stratification, and treatment decision-making in BCC. However, detailed and comprehensive bibliometric analyses in this field remain scarce.

**Methods:**

Publications related to AI and BCC were retrieved from the Web of Science Core Collection, Scopus, and Embase using predefined keyword strategies. All relevant records were exported, and 226 publications were ultimately included for analysis after screening and deduplication. Bibliometric analyses were performed using VOSviewer, CiteSpace, and the bibliometrix R package to characterize co-authorship networks, citations, keyword co-occurrence patterns, and journal distributions.

**Results:**

Annual publication output increased markedly after 2019, reaching 42 publications in 2025. The United States (43 publications) and China (36 publications) were the most productive countries, with the United States also hosting many of the leading institutions and authors. According to Bradford’s law of scattering, 13 core journals were identified; among them, Diagnostics (9 publications) and Skin Research and Technology (8 publications) had the highest output. Keyword analyses indicated that research hotspots center on deep learning–driven dermoscopic and digital pathology image analysis, primarily for classification and segmentation in computer-aided diagnosis of BCC.

**Conclusion:**

AI research in BCC has expanded rapidly since 2019. Future studies should prioritize multicenter, cross-device, and cross-population validation of multimodal AI systems and their integration into routine clinical practice to improve early detection and overall management of BCC.

## 1 Introduction

Basal cell carcinoma (BCC) is the most common skin cancer and accounts for approximately 85% of non-melanoma skin cancers (NMSC) (1). With population ageing and cumulative ultraviolet exposure, BCC incidence continues to rise, increasing healthcare burden (2, 3). Although metastasis is rare and the overall prognosis is generally favorable, delayed diagnosis may lead to extensive local tissue destruction and disfigurement (4). Early and accurate diagnosis is therefore critical to prevent local damage, improve patients’ quality of life, and optimize the allocation of healthcare resources. In routine practice, clinical diagnosis of BCC primarily relies on naked-eye examination and dermoscopy (5). Histopathological examination is mandatory for ambiguous lesions, ulcerated or large tumours with uncertain diagnosis, and also required for high-risk BCCs to evaluate surgical margins (6). However, diagnostic accuracy varies with expertise; lower sensitivity in primary care can lead to missed early cancers and unnecessary biopsies (7, 8). These limitations underscore an urgent need for objective, non-invasive, and scalable diagnostic tools.

Recently, artificial intelligence (AI)—particularly deep learning algorithms based on convolutional neural networks (CNNs), which can automatically learn complex visual patterns from images—has driven a rapid transformation in medical image analysis (9). In dermatology, AI models can learn from large repositories of clinical and dermoscopic images, automatically extracting subtle features that are difficult for the human eye to discern. This capability enables faster, more objective, and more accurate recognition and classification of skin lesions. Multiple studies show that AI systems can match or even surpass board-certified dermatologists in diagnosing malignant melanomas and carcinomas from benign nevi (10). Beyond classification, AI has also shown promise in delineating tumor margins, estimating recurrence risk, and supporting personalized treatment planning (11).

As research on AI in cutaneous oncology has expanded rapidly, it has become increasingly difficult to grasp the overall structure of the field, the leading contributors, and emerging research priorities (12). Bibliometrics analysis therefore provides a quantitative framework to map influential work, collaboration patterns, and emerging topics beyond narrative reviews (13, 14). Nevertheless, existing bibliometric studies in dermatologic AI have predominantly focused on melanoma, and to date no systematic bibliometric analysis has specifically examined AI research in BCC.

Therefore, clarifying how AI technologies have been applied to the diagnosis, treatment, and management of BCC is essential not only for improving the efficiency and accuracy of clinical care and promoting earlier detection of skin cancers, but also for advancing precision medicine and alleviating healthcare system pressures. The present study aims to provide a bibliometric and visual analysis of AI-related BCC research to characterize trends, hotspots, and future directions, thereby addressing a major evidence gap in this domain.

## 2 Materials and methods

### 2.1 Data collection and retrieval strategy

**Figure 1:**
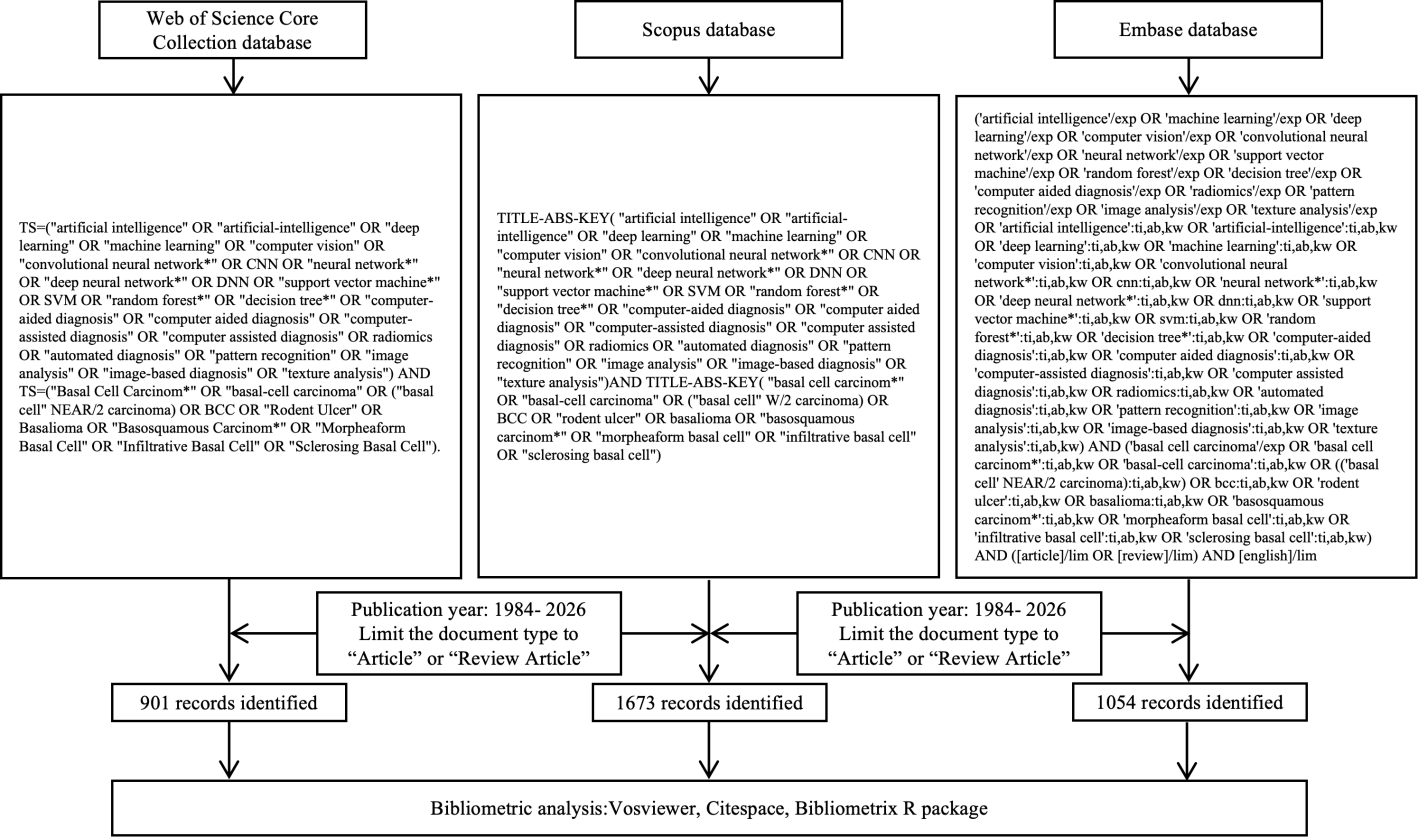
Data retrieval and analysis process.

To ensure comprehensive coverage and data accessibility, a systematic literature search was conducted in the Web of Science Core Collection (WoSCC), Scopus, and Embase. A common search framework combining AI-related terms (e.g., “artificial intelligence,” “machine learning,” “deep learning,” “computer vision,” and “neural network*”) with BCC-related terms (e.g., “basal cell carcinoma,” “basalioma,” and “rodent ulcer”) was adapted to the syntax and indexing system of each database. Specifically, the Topic (TS) field was used in WoSCC, TITLE-ABS-KEY in Scopus, and Emtree subject headings together with free-text terms in Embase. Additionally, database-specific controlled vocabulary was incorporated where appropriate. The detailed search strategy and screening workflow are presented in **Figure 1**.

After retrieval, records were restricted to English-language articles and reviews. The WoSCC, Scopus, and Embase searches yielded 901, 1,673 and 1,054 records, respectively (3,628 in total). After merging and deduplication, 1,963 records were retained and exported in plain-text format. Searching and data download were completed on 28 November 2025.

### 2.2 Data selection and standardization

Records were imported into Zotero for screening by title/abstract and, when needed, full-text review. Studies were excluded if: 1) not simultaneously addressing both AI and BCC; 2) non-BCC subject was investigated; or 3) non-AI techniques were employed. In total, 226 publications (200 articles and 26 reviews) were ultimately included in this study.

Before conducting the bibliometric analyses, we standardized the dataset to reduce bias arising from inconsistencies in author names and keyword expressions. All procedures were performed independently by two researchers, who then cross-checked the results. Any discrepancies were resolved through discussion with a third researcher until consensus was reached.

### 2.3 Bibliometric and visualization analysis

We utilized VOSviewer 1.6.20, CiteSpace 6.3.3, and the R package bibliometrix 5.2.0 to analyze publications on AI in BCC. To visualize the results, we employed Microsoft Excel 2024 and Scimago Graphica Beta 1.0.53. VOSviewer enables the construction and visualization of bibliometric networks and is well suited for processing large-scale datasets (15, 16). In this study, we used VOSviewer to analyze countries/regions, institutions, authors, journals, highly cited publications, and keywords. CiteSpace supports visual exploration of knowledge maps and can identify research trends and emerging topics through citation and burst analyses (17, 18). We applied CiteSpace to generate dual-map overlays of journals and to detect keyword bursts (19). The R package bibliometrix provides comprehensive bibliometric analysis functionality within the R environment and supports visualization of multiple types of networks and matrices (20). According to Bradford’s law of scattering (21), we identified core journals in AI-related BCC research and analyzed them using bibliometrix. Journal impact factors were obtained from the 2025 Journal Citation Reports (JCR).

## 3 Results

### 3.1 Basic quantitative analysis of information

A total of 3,628 records were initially retrieved from WoSCC, Scopus and Embase database. Following the screening process described above, 226 relevant publications (200 original articles and 26 reviews) were retained for analysis. The earliest article in this corpus addressing AI applications in BCC was published in 2004. Overall, related studies were published by researchers from 39 countries/regions, involving 1,472 authors affiliated with 504 institutions. These works were disseminated across 121 distinct journals. The corpus contained 870 unique keywords, including 535 Author’s Keywords (DE) and 335 Keywords Plus (ID). Collectively, the included publications cited 7,316 references published in 2,545 different journals.

### 3.2 Publication trends by year

**Figure 2:**
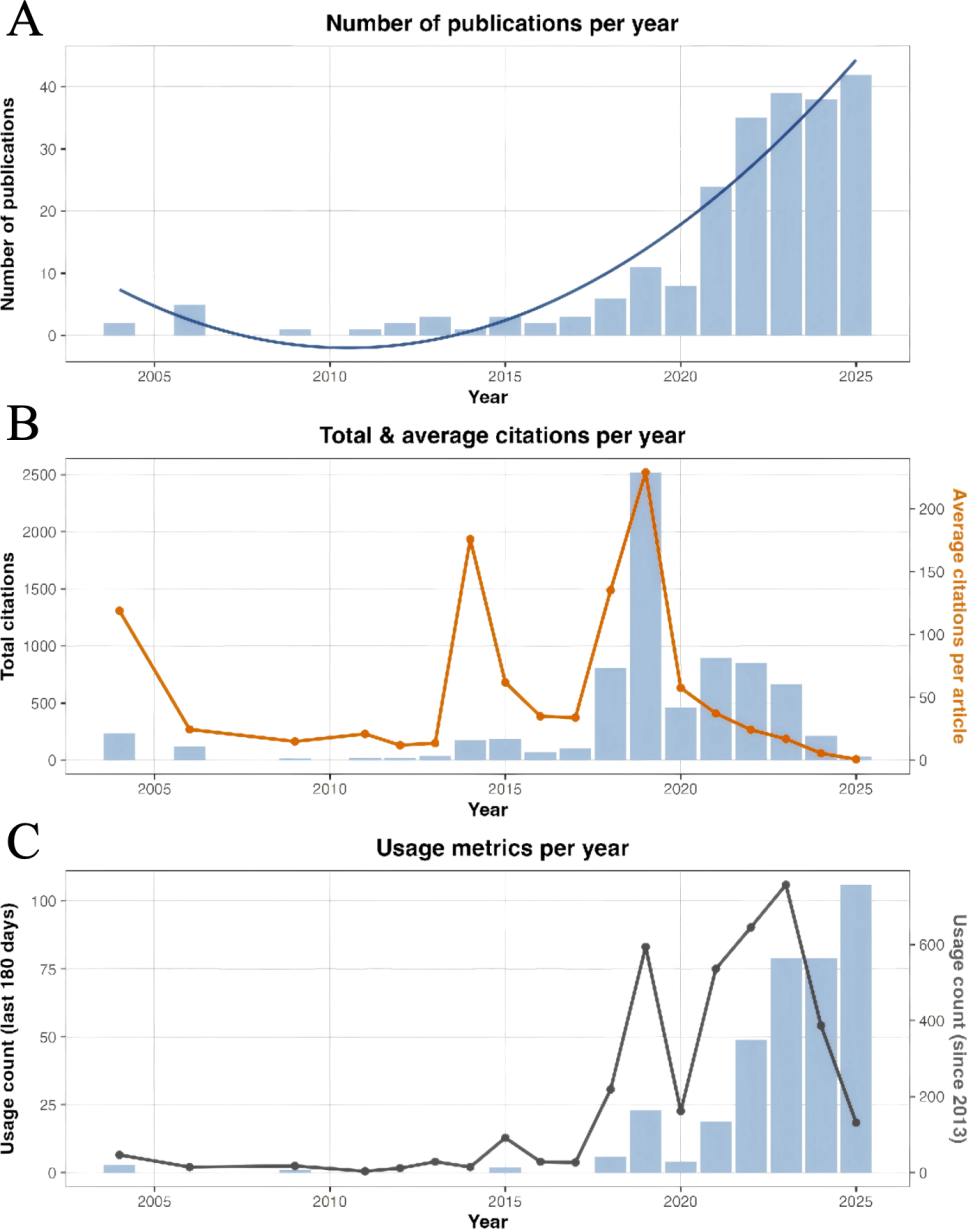
Yearly publication outputs in AI in basal cell carcinoma research. **(A)** Number of publications per year and growth trend of publications. **(B)** Total citations of publications per year and average citations per publication per year. **(C)** Number of publications usage count in the last 180 days per year and number of publications usage count since 2013 per year.

As shown in **Figure 2**, we plotted the annual publication trends of AI in BCC research. In terms of publication volume (**Figure 2A**), the early phase (2004–2015) was characterized by only 1–5 papers per year, indicating a sparse and exploratory stage. From 2015 to 2018, the number of publications increased gradually, reaching 6 papers in 2018 and marking the onset of a steady growth phase. Since 2019, the field has entered a rapid expansion phase: 11 and 8 papers were published in 2019 and 2020, respectively, followed by a marked increase to 24 papers in 2021. The annual output then rose further to 38 papers in 2024, and 42 in 2025, representing the current peak. The fitted curve shows an overall near-exponential growth pattern, indicating a pronounced acceleration of research activity in this domain.

In terms of citations volume (**Figure 2B**), the average number of citations per paper in this field is 32.95, with an average of approximately 6.05 citations per paper per year. The total citation curve exhibits several prominent peaks in the mid-term period, particularly around 2018–2020. Notably, the 2019 Nature Medicine article by Campanella et al. has been cited 1,616 times, while the 2018 Journal of Investigative Dermatology paper by Han et al. and the 2019 Lancet Oncology paper by Tschandl et al. have each received several hundred citations, substantially contributing to the total and average citation counts in their respective years. Overall, as the annual number of publications increases, the total citations continue to rise, whereas the average citations per paper show a slight decrease in the most recent years but remain at a relatively high level.

With respect to usage metrics (**Figure 2C**), both the annual cumulative usage counts and the usage in the last 180 days have increased markedly since 2018, with a series of peaks emerging after 2020 that broadly parallel the rapid growth in publication volume. The high recent usage of these papers, together with their strong citation performance, indicates that research on AI applications in the diagnosis and management of BCC is attracting growing attention and has become an important component of the broader NMSC research landscape.

### 3.3 Countries and regions

**Figure 3:**
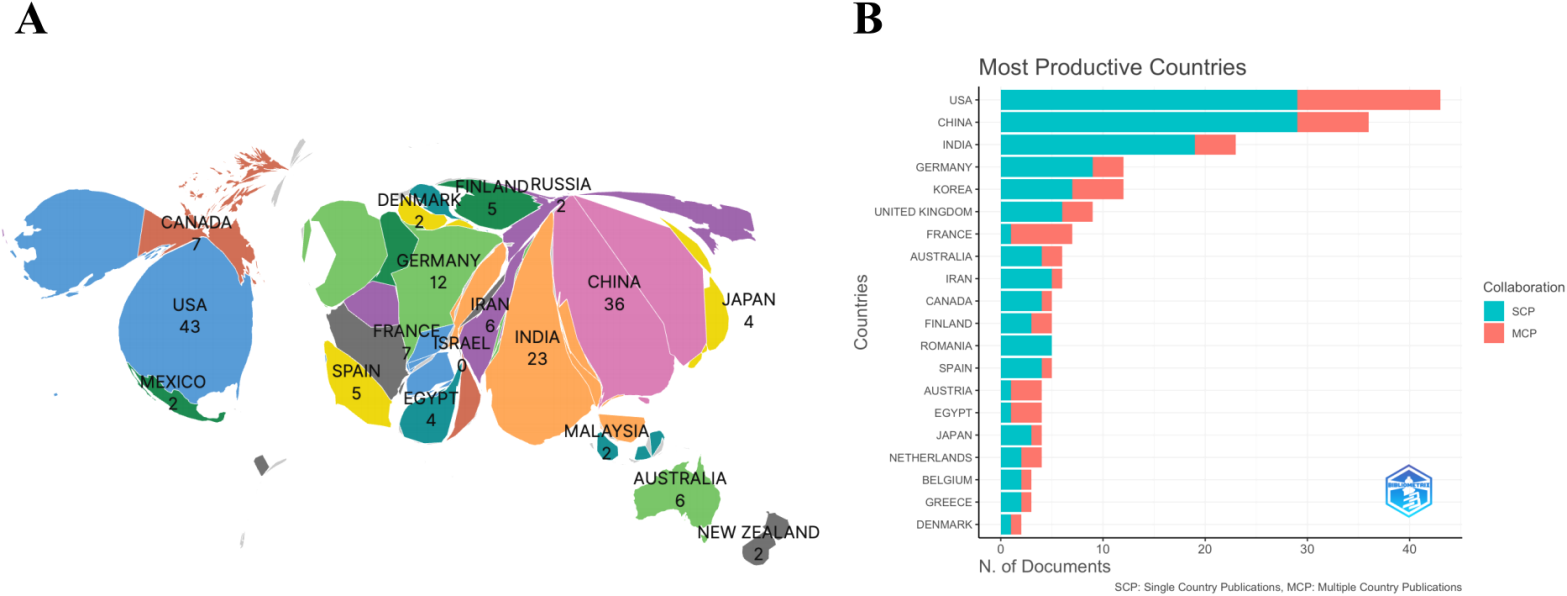
Geographic distribution corresponding authors ranked by number of publications in the field of AI in basal cell carcinoma research, based on bibliometric data: **(A)** Publications by countries/regions; **(B)** The top 20 countries/regions of corresponding authors ranked by number of publications. MCP, multiple country publications; SCP, single country publications.

**Figure 3A** depicts the global distribution of AI-related publications in BCC research. In terms of the number of publications by the corresponding authors’ countries, the United States ranked first (n = 43, 19.0%), followed by China (n = 36, 15.9%), India (n = 23, 11.5%), and Germany and South Korea (n = 12 each, 5.3%). Overall, research in this field remains largely driven by high-income countries, although several emerging economies are increasingly contributing to the literature.

As shown in **Figure 3B**, 30.53% of all articles involved international co-authorship. Collaboration patterns varied substantially across countries. Although the United States led in publication volume (n = 43), most of its papers were produced through single-country collaborations (SCP = 29, MCP_Ratio = 0.326). China ranked second in publication volume (n = 36) but exhibited a relatively lower rate of international collaboration (MCP_Ratio = 0.194), suggesting a greater reliance on domestic research networks. In contrast, France published only seven papers but achieved an international collaboration rate of 85.7% (MCP_Ratio = 0.857), showing a distinct “high collaboration–high connectivity” pattern and reflecting a tendency among some European countries to conduct research within multi-center, multi-national networks.

### 3.4 Institutions

We analyzed the publication output of 504 institutions. Among them, 355 institutions published only one publication, 87 institutions published two publications, 36 institutions published three publications, and 26 institutions each published between four and nine publications. The top 10 institutions by publication count are summarized in **Table 1**. Of these, four are based in the United States, two in Spain, and one each in South Korea, the United Kingdom, France, and Australia. Memorial Sloan Kettering Cancer Center (United States) had both the highest number of publications (n = 9) and the highest citation count (2,664 citations). Cornell University ranked second in total citations, with 1,727 citations.

**Table 1:** Top 10 institutions by number of publications on AI in basal cell carcinoma research.

Rank	Institution	Publications	Citations	Country
1	Memorial Sloan Kettering Cancer Center	9	2664	United States
2	Missouri University of Science and Technology	8	113	United States
3	University of Missouri System	8	161	United States
4	Sungkyunkwan University	7	390	South Korea
5	University of London	7	464	United Kingdom
6	Centre National De La Recherche Scientifique	6	396	France
7	Hospital Clinic De Barcelona	6	461	Spain
8	University of Barcelona	6	461	Spain
9	Cornell University	5	1727	United States
10	University of Queensland	5	917	Australia

**Figure 4:**
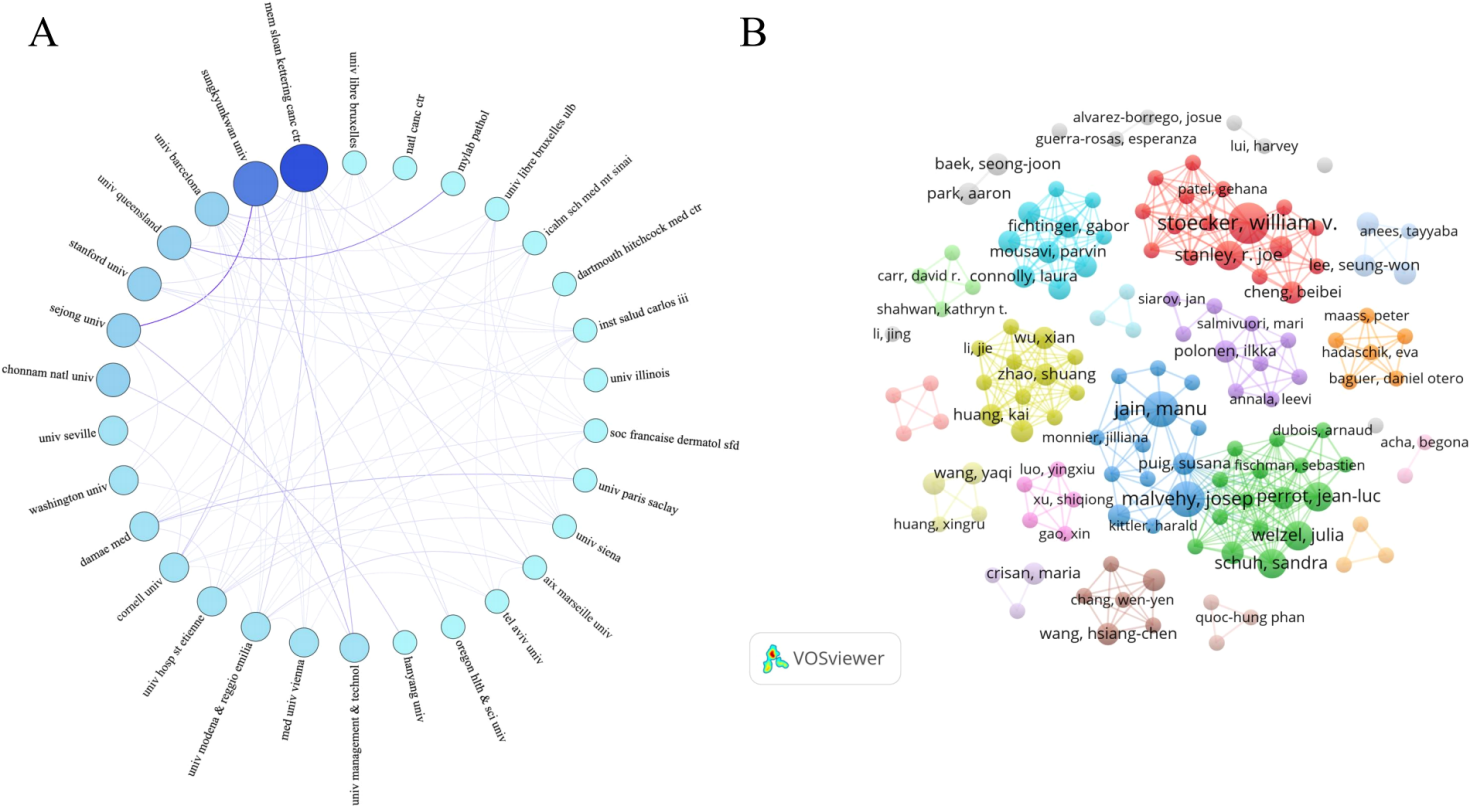
Networks of collaboration on AI in basal cell carcinoma: **(A)** Institutional collaboration. **(B)** Author collaboration.

To visualize inter-institutional collaboration, we constructed an institutional co-authorship network using the 30 most productive institutions (**Figure 4A**). In this network, node size is proportional to the number of publications produced by each institution, and nodes are displayed in shades of blue, with darker colors indicating higher output. Edges between nodes represent collaborative links, and thicker edges denote more frequent co-authorship. Overall, the leading institutions form a highly interconnected network, with dense collaborative ties among major centers in this field.

### 3.5 Authors

A total of 1,472 researchers were identified as authors of publications on AI in BCC. Among them, 1,307 authors published only one publication, 123 authors published two publications, 32 authors published three publications, 6 authors published four publications, and four authors published between five and seven publications. **Figure 4B** displays the collaboration network of the 136 authors who contributed two or more publications. In this co-authorship network, 25 distinct author clusters were identified. Most collaborations occurred within these clusters, although close links were also observed between several major groups.

**Table 2** lists the top 10 authors by publication count. Stanley (7 publications, 84 citations) and Stoecker (7 publications, 81 citations) can be regarded as the most prolific authors, with Malvehy (6 publications, 461 citations) following closely behind. Notably, Fuchs ranks 10th in publication count but has accrued 1,692 citations. The top 10 authors were distributed across six co-authorship clusters, with four authors belonging to the same cluster.

**Table 2:** Top 10 authors by publication output on AI in basal cell carcinoma research.

Rank	Author	Publications	Citations	Country
1	Stanley, Ronald. J.	7	84	United States
2	Stoecker, William V.	7	81	United States
3	Malvehy, Josep	6	461	Spain
4	Jain, Manu	5	86	United States
5	Tschandl, Philipp	4	478	Austria
6	Puig, Susana	4	460	Spain
7	Lee, Seung Won	4	200	Korean
8	Perrot, Jean-Luc	4	96	France
9	Schuh, Sandra	4	46	Germany
10	Fuchs, Thomas J.	4	1692	United States

### 3.6 Journals

**Figure 5:**
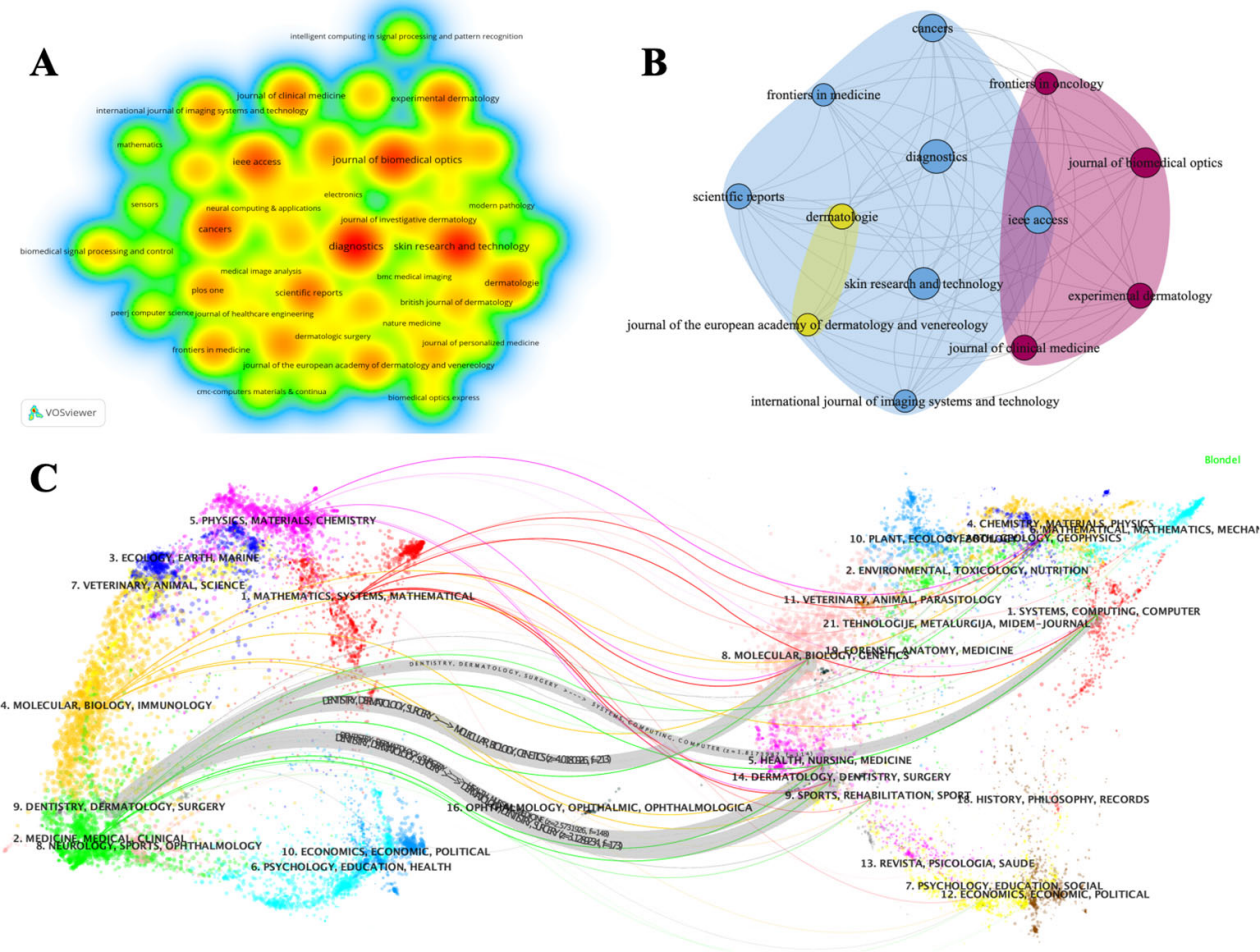
Analysis of journals related to AI in basal cell carcinoma research: **(A)** Map of journals with ≥ 2 publications; **(B)** Citation map of the 13 core journals identified by Bradford’s law of scattering; **(C)** Dual-map overlay analysis of journals.

The 226 publications included in this study were published across 121 different journals. Among these, 76 journals published only one publication, 20 journals published two publications, and 25 journals each published between three and nine publications. **Figure 5A** shows the 45 journals that published at least two publications. In this visualization, node size reflects the number of publications contributed by each journal, with larger nodes indicating higher output. Node color shifts toward red as publication volume increases.

Based on Bradford’s law of scattering, 13 core journals were identified, collectively publishing 72 publications. **Table 3** summarizes these journals’ publication counts, countries of origin, impact factors, and JCR quartiles. Diagnostics (Switzerland) ranked first with nine publications, followed by Skin Research and Technology (Denmark) with eight. Apart from Dermatologie (Germany), all core journals are classified in the Q1 or Q2 JCR quartiles. Among these journals, five are published in Switzerland, three in the United States, two in the United Kingdom, two in Denmark, and one in Germany. **Figure 5B** illustrates the citation network among these 13 journals.

**Table 3:** The 13 core research productivity journals identified by Bradford’s law of scattering.

Country	Journal	Publications	IF (2025)	JCR (2025)
Switzerland	Diagnostics	9	3.3	Q1
Denmark	Skin Research and Technology	8	3.2	Q1
United States	Journal of Biomedical Optics	7	2.9	Q2
Switzerland	Cancers	6	4.4	Q2
United States	IEEE Access	6	3.6	Q2
Germany	Dermatologie	5	0.7	Q4
Denmark	Experimental Dermatology	5	3.1	Q2
Switzerland	Journal of Clinical Medicine	5	2.9	Q1
United Kingdom	Scientific Reports	5	3.9	Q1
Switzerland	Frontiers in Medicine	4	3.0	Q1
Switzerland	Frontiers in Oncology	4	3.3	Q2
United States	International Journal of Imaging Systems and Technology	4	2.5	Q2
United Kingdom	Journal of the European Academy of Dermatology and Venereology	4	8	Q1

To further characterize the distribution of source and target journals, we employed a dual-map overlay visualization (**Figure 5C**). The left panel depicts the citing journals, whereas the right panel represents the cited journals. Labels indicate the dominant subject areas covered by each group of journals. Colored trajectories represent citation paths originating from the left (citing) map and pointing toward the right (cited) map. Four major citation paths are visible. All four originate from the “DENTISTRY, DERMATOLOGY, SURGERY” cluster. The first path leads to “SYSTEMS, COMPUTING, COMPUTER” (z = 1.8173987, f = 114); the second to “MOLECULAR, BIOLOGY, GENETICS” (z = 4.0180926, f = 213); the third to “HEALTH, NURSING, MEDICINE” (z = 2.5731926, f = 148); and the fourth terminates at “DERMATOLOGY, DENTISTRY, SURGERY” (z = 3.1289234, f = 173). This pattern reflects a bidirectional knowledge flow in which clinical dermatology journals frequently cite work in computational and basic science journals, with subsequent citations returning to clinical outlets.

### 3.7 Highly cited publications

**Table 4** lists the 10 most cited publications. Together, these publications have been cited 3,558 times, with an average of 355.8 citations per publication. The publication “Clinical-Grade Computational Pathology Using Weakly Supervised Deep Learning on Whole Slide Images,” published in Nature Medicine in 2019, received the highest number of citations (1,616). The second most cited publication, “Classification of the Clinical Images for Benign and Malignant Cutaneous Tumors Using a Deep Learning Algorithm,” was published in the Journal of Investigative Dermatology in 2018 and has been cited 412 times. Most of these highly cited publications appeared between 2018 and 2022, with individual citation counts ranging from 138 to 1,616.

**Table 4:** The top 10 cited publications.

Rank	Title	First author	Journal	Year	DOI	Total citations
1	Clinical-Grade Computational Pathology Using Weakly Supervised Deep Learning on Whole Slide Images	Campanella, Gabriele	Nature Medicine	2019	10.1038/s41591-019-0508-1	1616
2	Classification of the Clinical Images for Benign and Malignant Cutaneous Tumors Using a Deep Learning Algorithm	Han, Seung Seog	Journal of Investigative Dermatology	2018	10.1016/j.jid.2018.01.028	412
3	Comparison of the Accuracy of Human Readers Versus Machine-Learning Algorithms for Pigmented Skin Lesion Classification: An Open, Web-Based, International, Diagnostic Study	Tschandl, Philipp	Lancet Oncology	2019	10.1016/S1470-2045(19)30333-X	335
4	Detection Of Skin Cancer by Classification of Raman Spectra	Sigurdsson, S	IEEE Transactions on Biomedical Engineering	2004	10.1109/TBME.2004.831538	209
5	The Skin Cancer Classification Using Deep Convolutional Neural Network	Dorj, Ulzii-Orshikh	Multimedia Tools and Applications	2018	10.1007/s11042-018-5714-1	189
6	Skin Lesions Classification into Eight Classes for Isic 2019 Using Deep Convolutional Neural Network and Transfer Learning	Kassem, Mohamed A.	Ieee Access	2020	10.1109/ACCESS.2020.3003890	188
7	Update of the European Guidelines for Basal Cell Carcinoma Management Developed by The Guideline Subcommittee of The European Dermatology Forum	Trakatelli, Myrto	European Journal of Dermatology	2014	10.1684/ejd.2014.2271	176
8	Artificial Intelligence and Machine Learning Algorithms for Early Detection of Skin Cancer in Community and Primary Care Settings: A Systematic Review	Jones, O. T.	Lancet Digital Health	2022	10.1016/S2589-7500(22)00023-1	152
9	The Development of a Skin Cancer Classification System for Pigmented Skin Lesions Using Deep Learning	Jinnai, Shunichi	Biomolecules	2020	10.3390/biom10081123	143
10	Systematic Outperformance of 112 Dermatologists in Multiclass Skin Cancer Image Classification by Convolutional Neural Networks	Maron, Roman C.	European Journal of Cancer	2019	10.1016/j.ejca.2019.06.013	138

Analysis of the top 10 highly cited publications indicates that their primary focus is on AI applications for the diagnosis of BCC and related skin tumors. Seven publications concentrate on deep learning–based image classification, employing convolutional neural network or weakly supervised deep learning models to classify whole-slide images, clinical photographs, and dermoscopic images into benign versus malignant lesions or multiple tumor categories, and to compare performance against dermatologists’ diagnoses (22–27). In addition, one highly cited publication used Raman spectroscopy combined with machine learning to classify skin cancers, exploring “optical biopsy” or non-invasive diagnostic pathways (28). These studies primarily rely on physical or spectral signals as input features, complementing image-driven deep learning approaches. The remaining two publications comprise an updated guideline on BCC management and a systematic review of AI/machine learning for early skin cancer screening in community and primary care settings (29, 30). Overall, the highly cited literature suggests that current research in this field is predominantly oriented toward deep learning–driven skin tumor image recognition, supplemented by spectroscopic, non-invasive diagnostic strategies and the synthesis of clinical guidelines and evidence.

### 3.8 Keywords

**Figure 6:**
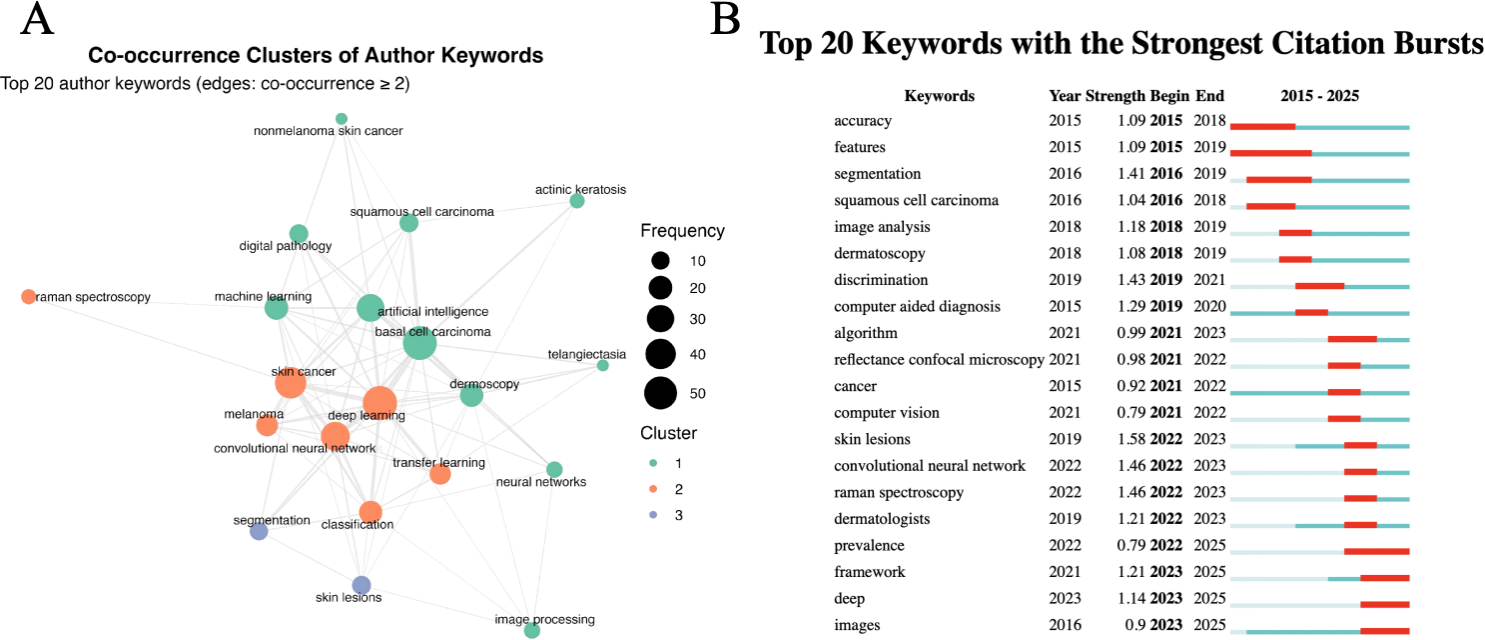
Bibliometric analysis of keywords on AI in basal cell carcinoma research: **(A)** Keyword co-occurrence network. **(B)** Top 20 keywords with the strongest citation bursts.

Keywords are commonly used to highlight major research themes and to represent the core content of publications. Frequently occurring keywords typically correspond to hot topics within a field. **Table 5** presents the top 20 keywords related to AI in BCC research. Keywords that appeared at least five times were included in a cluster analysis, which identified three clusters (**Figure 6A**). Cluster 1 (orange) contains high-frequency keywords such as “deep learning,” “skin cancer,” and “neural networks.” Cluster 2 (green) includes keywords such as “basal cell carcinoma,” “artificial intelligence,” and “machine learning.” Cluster 3 (blue) comprises keywords such as “skin lesions” and “segmentation.”

**Table 5:** The 20 most frequently co-occurring author keywords.

Rank	Keywords	Frequency	Rank	Keywords	Frequency
1	deep learning	56	11	digital pathology	11
2	basal cell carcinoma	54	12	Skin lesions	11
3	skin cancer	44	13	squamous cell carcinoma	11
4	convolutional neural network	35	14	segmentation	10
5	artificial intelligence	31	15	image processing	8
6	machine learning	20	16	neural networks	8
7	classification	19	17	actinic keratosis	7
8	dermoscopy	19	18	raman spectroscopy	7
9	melanoma	16	19	nonmelanoma skin cancer	6
10	transfer learning	15	20	telangiectasia	6

Keyword burst analysis was used to identify abrupt increases in keyword frequency over specific time periods. This approach can be used to detect emerging research frontiers and to track shifts in research hotspots over time. As shown in **Figure 6B**, the top five keywords ranked by burst strength were “skin lesions” (intensity = 1.58, 2022–2023), “convolutional neural network” (intensity = 1.46, 2022–2023), “Raman spectroscopy” (intensity = 1.46, 2022–2023), “discrimination” (intensity = 1.43, 2019–2021), “segmentation” (intensity = 1.41, 2016–2019), and “computer-aided diagnosis” (intensity = 1.29, 2019–2020).

## 4 Discussion

This bibliometric analysis of 226 publications on AI in BCC identified leading countries, academic clusters, and key authors; traced the field’s developmental trajectory; and delineated research hotspots and future directions.

Since 2019, publications on AI in BCC have increased markedly, peaking in 2025. In parallel, total citation counts, citations within the last 180 days, and citations since 2013 have all risen substantially after 2019, mirroring output growth. Peaks in annual total citations and citations per publication in specific years indicate that attention in this field has been driven by the clustered emergence of a small number of highly influential papers, a diffusion pattern commonly observed in emerging interdisciplinary domains. Several landmark studies published between 2018 and 2019 likely explain this shift. Notably, 2019 appears to mark a turning point. A previous study demonstrated that deep learning could classify clinical images of benign and malignant cutaneous tumors with high accuracy (31). Subsequent studies represented pivotal milestones in deep learning-based clinical image classification with explicit human–machine comparisons, shifting the narrative from algorithmic feasibility to clinical comparability (23, 27). In parallel, a 2019 Nature Medicine study established a scalable, generalizable paradigm for tumor pathology AI by achieving clinical-grade computational pathology on whole-slide images (WSIs) using weakly supervised deep learning, providing a key foundation for the rapid rise of computational and digital pathology in skin tumors (22). These publications suggest 2019 was a watershed “proof-of-concept” year, catalyzing subsequent field expansion.

At the global level (based on the corresponding author’s country), the United States and China lead in publication volume, followed by India, Germany, and South Korea. The leadership of the United States and China reflects substantial investment and robust R&D capacity in this interdisciplinary area, likely sustained by targeted governmental funding, mature research ecosystems, and strong academic–industry linkages. Their collaboration patterns, however, differ markedly. While maintaining high output, the United States demonstrates substantial multinational collaboration (SCP = 29, MCP = 14, MCP_Ratio = 0.326), underscoring its central position in international networks. By contrast, China’s production is predominantly domestically driven (SCP = 29, MCP = 7, MCP_Ratio = 0.194), with India showing a similar pattern (SCP = 19, MCP = 4, MCP_Ratio = 0.174). Notably, France shows strong reliance on international collaboration despite its modest output (SCP = 1, MCP = 6, MCP_Ratio = 0.857), as does South Korea (MCP_Ratio = 0.417). At the institutional level, U.S. centers dominate institutionally, with five of the top ten institutions based in the United States; Memorial Sloan Kettering Cancer Center and Missouri University of Science and Technology are particularly prominent. This clustering of high-impact institutions likely reflects the United States’ long-standing investment in oncology research, highly integrated public research infrastructure, and strategic funding schemes.

These collaboration patterns have direct clinical implications. AI model generalizability is strongly influenced not only by imaging equipment and acquisition protocols, but also by population characteristics such as age, sex, skin phototype, lesion site, and disease spectrum. Variation in these factors can alter lesion appearance, background skin features, case mix, and disease prevalence, thereby affecting model calibration, sensitivity, specificity, and error patterns across populations. Cross-center datasets are therefore critical for robust validation and building models that generalize across diverse environments (32, 33). Countries with extensive networks are better positioned to develop broadly applicable frameworks.

Author productivity follows Lotka’s law (34), showing pronounced skewness: 88.79% of authors contributed only a single publication, whereas a small core group (4 authors; 0.27%) authored five or more. The dominance of U.S.-based groups is again evident, with four of the ten most prolific authors belonging to the same collaborative cluster. This collaborative cluster appears to center on image-based diagnostic AI for BCC and related skin tumors, spanning dermoscopic and clinical image analysis, advanced optical imaging, ex vivo microscopy, and pathology-oriented decision-support applications (35–40). This pattern suggests that one of the most productive collaborative streams in the field is oriented toward clinically deployable, image-based AI across multiple diagnostic modalities rather than a single narrow technical track. However, this high productivity coexists with a fragmented network structure, suggesting specialization within relatively insular clusters at the expense of cross-cluster knowledge exchange—potentially constraining interdisciplinary innovation. The convergence of concentration at geographic, institutional, and author levels highlights systemic inequalities in resource allocation and scientific recognition. The United States’ “triple dominance” (country–institution–author) underscores path dependence within the research ecosystem. The contrast between more self-reliant models (China, India) and collaboration-dependent systems (France, South Korea) reflects divergent strategies shaped by funding policies and institutional capacity. Importantly, while such research silos can enable rapid progress, they may hinder translational breakthroughs by increasing reliance on localized and relatively homogeneous datasets, thereby amplifying algorithmic bias and limiting the generalizability of AI models across different populations, ethnic groups, countries, and clinical settings. In addition, fragmented research structures, partly shaped by differences in funding support and policy implementation, may impede the large-scale, multicenter external validation required for clinical translation. Future initiatives should incentivize cross-cluster collaboration.

Publications are distributed across 121 journals, with more than half (76 journals) publishing only a single paper, underscoring the highly interdisciplinary and still-emerging nature of AI applications in BCC. Bradford’s law identified 13 core journals that concentrate much of the literature, partially mitigating this fragmentation. These core journals are predominantly based in Switzerland (n = 5), the United States (n = 3), Denmark and the United Kingdom (n = 2 each), and most are indexed in the JCR Q1 or Q2 quartiles, indicating integration into established and reputable publication venues. The prominence of dermatology journals such as the Journal of the European Academy of Dermatology and Venereology and Experimental Dermatology, alongside specialized outlets in biomedicine, artificial intelligence, and optical engineering, reflects the convergence of diverse expertise. Dual-map overlay analysis further confirms this multidisciplinarity, revealing substantial citation flows and feedback loops between clinical DERMATOLOGY, SURGERY domains and MOLECULAR, BIOLOGY, GENETICS methodology domains.

The keyword landscape further illuminates the core themes and dynamic evolution of AI research in BCC. Bibliometric analysis of author keywords highlights both current hotspots and emerging frontiers. The co-occurrence network links “basal cell carcinoma” and “artificial intelligence” closely with “dermoscopy,” “digital pathology,” “nonmelanoma skin cancer,” “squamous cell carcinoma,” and “actinic keratosis,” suggesting that BCC is frequently positioned as a key class within broader NMSC/skin tumor differential-diagnosis frameworks. This aligns with the prominence of multiclass classification and human–machine comparison studies among the highly cited publications in **Table 4**. Methodologically, keywords such as”deep learning,” “convolutional neural network,” “classification,” and “transfer learning”form the backbone of the field, indicating that image-based AI classification remains the dominant research direction in automated lesion analysis. CNNs have reached dermatologist-level performance in dermoscopic classification, and several studies report that AI algorithms can outperform human experts in BCC detection (41–43). Han et al. (31) demonstrated high-accuracy classification of benign and malignant cutaneous tumors from clinical images, with performance comparable to that of dermatologists. Tschandl et al. (23) conducted an open, international web-based diagnostic study that directly compared machine-learning algorithms with human readers. Maron et al. (27) further showed, under controlled conditions, that CNNs consistently outperformed 112 dermatologists in multiclass skin cancer image classification tasks. More specific evidence is also available for BCC-related tasks. Huang et al. (44) compared a CNN model with 21 dermatologists in distinguishing BCC from seborrheic keratosis and reported expert-level model performance. Mei et al. (43) further demonstrated in a prospective study that deep learning assistance could improve dermatologist performance in this classification task. Key advances include encoder–decoder architectures for automated lesion segmentation, enabling more precise isolation of regions of interest for downstream analysis (45, 46). Multiclass frameworks further support discrimination across a broad spectrum of skin tumors and benign versus malignant lesions with high sensitivity and specificity. Meanwhile, the rise of “segmentation” and clinically oriented digital pathology workflows indicates a shift toward outputs that are more actionable in routine practice.

Keyword burst analysis adds a temporal dimension. Early bursts emphasized feasibility and performance (“accuracy”, “features”, “segmentation” and “dermoscopy”), reflecting proof-of-concept work. More recent bursts shifted toward real-world context, framework-level design, and scaled data (“algorithm”, “computer vision”, “reflectance confocal microscopy”, “prevalence”, “deep” and “images”). This pattern likely reflects an expansion from dermoscopy-centered feasibility studies toward broader image-based and multimodal research frameworks spanning clinical photography, dermoscopy, optical imaging, and pathology images. Overall, the frontier appears to be moving from demonstrating that AI can work to establishing robust and safe deployment across varying prevalence settings and clinical environments, with increasing emphasis on framework-level design, modality expansion, and robustness in deployment.

Looking ahead, AI research in BCC is more likely to converge than to proceed along isolated parallel tracks. Image classification, segmentation, digital pathology, and optical biopsy-related modalities are increasingly being integrated with clinical data and workflow-level decision support, rather than developed as entirely separate streams. This convergence is likely to be more clinically relevant than single-modality model development alone (47).

Clinically, given BCC’s high incidence, low metastatic potential, and strong surgical orientation, the value of AI should extend beyond binary benign–malignant discrimination to support key decision nodes along the care pathway. These include triage/referral, biopsy strategy optimization, margin and subtype risk alerts, and preoperative planning for Mohs micrographic surgery and excision–reconstruction, as well as follow-up and longitudinal surveillance, including serial assessment of equivocal lesions, post-treatment monitoring, and recurrence-oriented risk evaluation. Enabling these use cases requires moving from generic multiclass classification to structured, decision-linked outputs: localizable and reliable boundaries, morphologic markers of infiltrative or high-risk subtypes, and risk stratification for highly invasive or high-recurrence subgroups. The primary barriers to translation are less architectural than evidentiary and data-related. Dermoscopic and clinical image datasets commonly exhibit spectrum and verification biases, with biopsy-confirmed malignant-suspect lesions overrepresented and atypical or ambiguous cases underrepresented. In pathology, weak labeling and intra-slide heterogeneity are major constraints; whole-slide models trained only on slide- or case-level labels risk learning scanning artifacts or processing patterns rather than true tumor morphology. Future high-quality studies should therefore incorporate data-generation mechanisms and labeling strategies explicitly into methodological design.

Finally, as BCC becomes a defined target within broader differential-diagnosis frameworks, future work will likely emphasize end-to-end systems rather than isolated models. Such systems span front-end multimodal acquisition and quality control, mid-stream multiclass risk stratification, and back-end closed-loop integration with pathology and surgical workflows. This systems-level perspective is consistent with the rise of “framework-oriented” themes in our keyword analysis: deployable AI for BCC is not a single high-performing classifier, but a clinically embedded decision-support infrastructure that operates robustly across institutions and devices while meeting regulatory, interpretability, and audit requirements.

Taken together, current knowledge suggests that AI research in BCC has progressed beyond early proof-of-concept image classification and entered an early translational stage. Nevertheless, broader clinical application still depends on more consistent external validation, more diverse datasets, and closer integration with real-world clinical workflows.

## 5 Limitations

This study has certain limitations. Although we expanded retrieval beyond WoSCC by additionally searching Scopus and Embase, the analysis was restricted to English-language records, which may have excluded relevant evidence published in other languages or indexed in additional databases. We restricted the analysis to articles and reviews, excluding conference proceedings. Although author names and keywords were standardized, fragmentation bias can only be mitigated, not eliminated. Citation-based indicators have inherent constraints: highly cited publications are not necessarily the most rigorous, and recent studies may be underrepresented due to insufficient citation time. The rapid evolution of AI means our analysis captures only a time-bound snapshot.

## 6 Conclusion

This bibliometric analysis systematically delineates the evolving landscape of AI in BCC research. Since 2019, the field has undergone transformative growth, with landmark studies demonstrating that AI can achieve diagnostic accuracy comparable to dermatologists. The United States and China account for most research output, with the US serving as a collaboration hub and hosting leading institutions including Memorial Sloan Kettering Cancer Center and Missouri University of Science and Technology. Current research hotspots focus on deep learning-driven lesion classification and digital pathology, alongside integration of non-invasive modalities such as RCM and Raman spectroscopy. Future priorities include multi-center external validation to strengthen cross-device and cross-population generalizability, and deeper AI-clinician collaboration to overcome translation barriers. This bibliometric mapping provides a reference framework for designing translational studies aimed at earlier BCC detection, more precise surgical planning, and improved clinical outcomes.
